# Differential Regulation of the PGC Family of Genes in a Mouse Model of *Staphylococcus aureus* Sepsis

**DOI:** 10.1371/journal.pone.0011606

**Published:** 2010-07-15

**Authors:** Timothy E. Sweeney, Hagir B. Suliman, John W. Hollingsworth, Claude A. Piantadosi

**Affiliations:** 1 Department of Pathology, Duke University Medical Center, Durham, North Carolina, United States of America; 2 Department of Anesthesiology, Duke University Medical Center, Durham, North Carolina, United States of America; 3 Department of Medicine, Duke University Medical Center, Durham, North Carolina, United States of America; National Institute of Allergy and Infectious Diseases, National Institutes of Health, United States of America

## Abstract

The PGC family of transcriptional co-activators (PGC-1α [*Ppargc1a*], PGC-1β [*Ppargc1b*], and PRC [*Pprc*]) coordinates the upregulation of mitochondrial biogenesis, and *Ppargc1a* is known to be activated in response to mitochondrial damage in sepsis. Therefore, we postulated that the PGC family is regulated by the innate immune system. We investigated whether mitochondrial biogenesis and PGC gene expression are disrupted in an established model of *Staphylococcus aureus* sepsis both in mice with impaired innate immune function (TLR2^−/−^ and TLR4^−/−^) and in wild-type controls. We found an early up-regulation of *Ppargc1a* and *Ppargc1b* post-infection (at 6 h) in WT mice, but the expression of both genes was concordantly dysregulated in TLR2^−/−^ mice (no increase at 6 h) and in TLR4^−/−^ mice (amplified at 6 h). However, the third family member, PRC, was regulated differently, and its expression increased significantly at 24 h in all three mouse strains (WT, TLR2^−/−^, and TLR4^−/−^). *In silico* analyses showed that *Ppargc1a* and *Ppargc1b* share binding sites for microRNA mmu-mir-202-3p. Thus, miRNA-mediated post-transcriptional mRNA degradation could account for the failure to increase the expression of both genes in TLR2^−/−^ mice. The expression of mmu-mir-202-3p was measured by real-time PCR and found to be significantly increased in TLR2^−/−^ but not in WT or TLR4^−/−^ mice. In addition, it was found that mir-202-3p functionally decreases *Ppargc1a* mRNA *in vitro*. Thus, both innate immune signaling through the TLRs and mir-202-3p-mediated mRNA degradation are implicated in the co-regulation of *Ppargc1a* and *Ppargc1b* during inflammation. Moreover, the identification of mir-202-3p as a potential factor for *Ppargc1a* and *Ppargc1b* repression in acute inflammation may open new avenues for mitochondrial research and, potentially, therapy.

## Introduction

The PPAR-gamma coactivator (PGC) family of transcriptional co-activators have been called ‘master regulators’ of mitochondrial biogenesis because they co-activate the transcription factors nuclear respiratory factor-1 and -2 (NRF-1 (*Nrf1*), NRF-2 (*Gabpa*)), thereby initiating nuclear gene expression for mitochondrial proteins [1], [2]. The known PGC family gene products (PGC-1α (*Ppargc1a*), PGC-1β (*Ppargc1b*), and PRC (*Pprc*)) are variably affected by inflammation; for instance, *Ppargc1a* mRNA levels may decrease after exposure of cells to LPS, while PGC-1α protein content and stability are increased in mice exposed to LPS [3], [4], [5].

*Ppargc1a* levels in mice infected with *Staphylococcus aureus* (*S. aureus*) have been shown to increase as mitochondrial damage develops under the stresses of excessive reactive oxygen and nitrogen species generation [6], [7]. Mitochondrial damage in sepsis disrupts oxygen homeostasis, leading to a state of high tissue oxygenation but low tissue oxygen utilization sometimes called cytopathic hypoxia [8], [9]. Increased mitochondrial damage and cytopathic hypoxia are correlated with a high mortality rate [10]. Sepsis is a growing health problem and a leading cause of death in the U.S., and despite aggressive intensive care, still has an in-hospital mortality rate of approximately 30% [11].

The program of mitochondrial biogenesis is responsible for maintaining adequate mitochondrial mass and quality in the cell, and it is indispensable for energy homeostasis and cell viability during periods of cell stress that increase mitochondrial turnover. Mitochondrial biogenesis is a bi-genomic process that requires coordination between nuclear- and mitochondrial-encoded genes [12]. The cell activates mitochondrial biogenesis in response to sepsis, which helps to counteract the effects of mitochondrial damage and to maintain appropriate oxygen metabolism and ATP availability.

The signal transduction pathways that link sepsis-induced inflammation to the up-regulation of mitochondrial biogenesis are not well understood, but there is some evidence that the Toll-like receptor (TLR) family of innate immune receptors may provide such a link. The TLRs are transmembrane proteins that sense conserved pathogen-associated molecular patterns. When activated, the TLRs signal through various downstream kinases to mobilize pro-inflammatory transcription factors such as NF-κB, AP-1, and IRF3/7 [13]. TLR2 is activated by components of Gram-positive bacteria, while TLR4 is activated by components of Gram-negative bacteria [14]. It has been shown that TLR4^−/−^ mice, compared with WT mice, show less mitochondrial damage in a model of heat-killed *E. coli* sepsis, but also show less activation of mitochondrial biogenesis and a slower recovery of mtDNA copy number [15]. Thus, TLR signaling may directly link the innate immune response in sepsis to the regulation of mitochondrial biogenesis.

To better understand the regulatory role of the PGC family in the complex process of mitochondrial biogenesis during the acute inflammatory response, we used an established fibrin-clot model of *S. aureus* sepsis to test the hypothesis that TLR signaling leads to downstream regulation of the PGC family of genes. We examined the expression of genes of mitochondrial biogenesis program in this sepsis model in the livers of WT, TLR2^−/−^, and TLR4^−/−^ mice in order to compare the wild-type response to the effects of specific innate immune receptor deficiencies on mitochondrial biogenesis. This study focused on the liver because it is both a key metabolic and immune organ (through the presence of Kupffer cells), and because the liver receives the portal circulation and so its immune cells are stimulated by peritoneal infection.

Most of the research on the regulation of mitochondrial biogenesis has focused on the roles of cytosolic kinases and transcription factors that activate and regulate the genes involved, but post-transcriptional mechanisms are probably also important. In fact, several miRNAs have been demonstrated to target genes involved in metabolism, including PGC-1α [16], [17]. Such miRNAs are the products of non-protein-coding genes that are processed into mature 19–21 bp sequences. For mRNA binding, the proximal 7–8 bp of the miRNA (the seed region) binds to a complimentary sequence in the 3′ UTR of a target mRNA, resulting in either sequestration or degradation [18]. Each miRNA is complementary to hundreds of mRNAs *in silico*, though comparatively few matches have been shown to result in gene silencing. Thus, along with gene promoter maps, we compared the 3′ UTRs of PGC family genes to locate conserved miRNA binding sites.

Our findings demonstrate a dampening and a delay in mitochondrial biogenesis during Gram-positive inflammation in both TLR-deficient mouse strains, but also demonstrate that *Ppargc1a* and *Ppargc1b* are co-regulated independently of PRC. Moreover, we report that *Ppargc1a* and *Ppargc1b* gene expression is negatively correlated with mir-202-3p, which is differently expressed in WT, TLR2^−/−^, and TLR4^−/−^ mice.

## Materials and Methods

### Mouse studies

The studies were conducted in C57Bl/6J mice purchased from Jackson Laboratories (Bar Harbor, ME) and in TLR2^−/−^ and TLR4^−/−^ mice on a C57Bl/6J background obtained from Shizuo Akira, Japan [14], [19], and backcrossed >10 generations onto the C57Bl/6J background. Mice of either gender weighing 20–30 grams were used under study protocol A262-07-09, which was approved by the Institutional Animal Care and Use Committee.

Mice were anesthetized with an intraperitoneal injection of xylazine and ketamine, and the abdomen was shaved and cleaned with povidone-iodine. Midline laparotomy was performed and an infected fibrin clot was inserted in the peritoneum. The peritoneum and abdomen were then closed with proline sutures. All mice were resuscitated with 1 ml of 0.9% NaCl administered subcutaneously. Mice were sacrificed at 6, 24, 48, or 72 hours PI by overexposure to isoflurane. Livers of healthy control (HC) mice of each strain were also obtained. The livers were harvested immediately and either the mitochondria were isolated at once or the tissue was snap-frozen and stored at −80°C.

To prepare the fibrin clots, *Staphylococcus aureus* (ssp aureus) was reconstituted and suspended in fibrin according to published methods [6]. The bacteria were sterilely inoculated on agar slants for 18 hours and then resuspended to a concentration of 10^10^cfu/ml based on optical density at 550 nm. Doses of 10^5^, 10^6^, or 10^7^ cfu were then suspended in 500 ul fibrin clots.

### Cell Studies

Mouse AML12 hepatocytes were purchased from the American Type Culture Collection (Manassas, VA). AML12 cells were cultured in 5% CO2–95% air at 37°C in DMEM/F12 medium (GibcoBRL, Grand Island, NY) containing l-glutamine and 2.438 g/L sodium bicarbonate. The medium was supplemented with 10% FBS, a mixture of insulin, transferrin, selenium (ITS; Sigma, St. Louis, MO) and 40 ng/ml dexamethasone.

AML12 cells were exposed to 10^7^ cfu heat-killed *S. aureus* per ml (prepared from the same strain as that implanted in mice) and were harvested at different time-points. Gene expression was tested by real time RT-PCR.

AML12 cells were transfected at approximately 60% confluency with scrambled siRNA (AllStars Negative Control siRNA, Qiagen) or miRNA mimic for mmu-mir-202-3p (Qiagen) using Lipofectamine RNAiMax transfection reagent (Invitrogen). Transfection to 80% was confirmed with BlockIT fluorescent oligo (Invitrogen). Serum starvation was achieved 24 hours after transfection by replacing cell culture media with media without FBS for 4 hours. mRNA was extracted with Trizol and gene expression was tested by real time RT-PCR.

### Mitochondria and mtDNA Isolation

Liver mitochondria were isolated from ∼2 g of fresh tissue using a modified Clark protocol [20]. Briefly, the livers were hand-dounced in a 0.25 M sucrose buffer and then centrifuged at 2,000x*g* for 3 min at 4°C. The supernatant was centrifuged at 12,500x*g* for 8 min at 4°C, and the pellet recovered for mtDNA extraction using the mtDNA Extraction Kit (Wako Chemical, Japan) according to the manufacturer's instructions.

### Real-Time RT-PCR

RNA was extracted from frozen liver with TRIzol reagent (Invitrogen, Oslo, Norway) and subjected to reverse transcription with the ImProm-II Reverse Transcription System (Promega, Madison, WI) according to the manufacturer's instructions. Mouse-specific primers were designed (Table S1) and real-time PCR was carried out in triplicate as described, using 18 s primers for internal controls [15]. Real-time PCR output for HC mice of each strain was set to one, and the relative quotients at later time points are shown. The mtDNA copy number count was determined in reference to a standard cytochrome *b* (Cyt *b*) plasmid with a Cyt *b* probe as described [15].

### MicroRNA PCR

MicroRNAs were prepared with an All-in-One™ miRNA qRT-PCR Detection Kit (GeneCopoeia, Rockville, MD) according to the manufacturer's instructions. Briefly, the extracted RNA was reverse-transcribed in the presence of a Poly-A polymerase with an oligo-dT adaptor. Quantitative PCR was then carried out with SYBR green detection with a forward primer for the mature miRNA sequence and a universal adaptor reverse primer.

### *In silico* analyses

To prepare promoter maps, mouse (*Mus musculus* NCBI assembly m37) and human (*Homo sapiens* NCBI assembly GRCh37) genomes were accessed on Ensembl (www.ensembl.org), and aligned using zPicture (zpicture.dcode.org). The alignments were then fed into rVista 2.0 (rvista.dcode.org) and analyzed for transcription factor consensus sequences according to the Transfac Professional library (v10.2) with similarities optimized for function [21].

MicroRNA binding predictions were made with TargetScan Mouse release 5.1 (www.targetscan.org/mmu_50) [22]. Specific microRNA binding patterns were then predicted on microRNA.org (www.microrna.org). mRNA folding and single-strand frequency predictions were made with mfold version 3.2 [23], [24], using mRNA sequences from the Ensembl database.

### Statistics

All grouped data are presented as means ± SD. The n values indicated in the figure legends are for the total number of mice from each strain. Each time point in the real-time PCR experiments was compared to the healthy control (HC) of its own strain using a one-sided Student's *t*-test. In addition, the 6 h time points between strains were compared using two-sided Student's *t*-tests. The level of significance for all tests was set at *P*<0.05.

## Results

### Characteristics of the Sepsis Model

**Figure 1:**
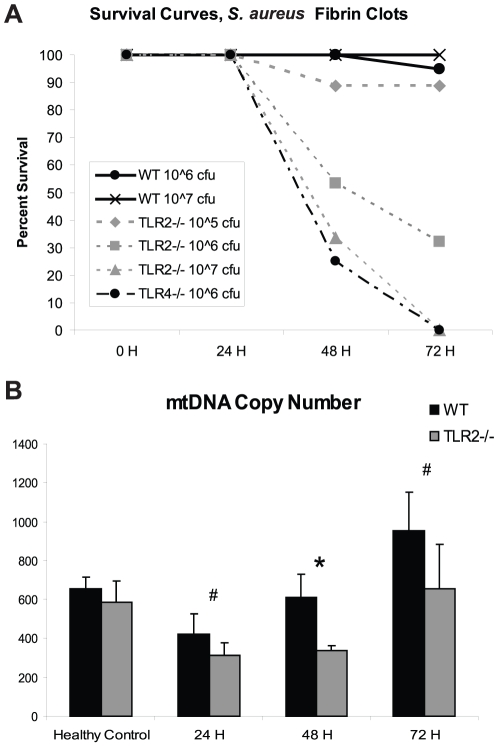
Survival curves and mtDNA content in *S. aureus* sepsis. (A) Survival curves for WT, TLR2^−/−^, and TLR4^−/−^ mice are shown. Doses of 10∧5, 10∧6, and 10∧7 are shown for TLR2^−/−^ mice, showing dose-response effects. (B) Absolute mtDNA content in WT and TLR2^−/−^ was measured by Q-PCR of Cyt *b* in comparison to known standard. TLR2^−/−^ mtDNA content is lower than WT mtDNA content in *S. aureus* sepsis at each time point (n = 4 at each time point; #, p = 0.05; *, p<0.01).

The dose-response behavior to *S. aureus* sepsis was evaluated in all three strains of mice. At all bacterial doses, WT mice showed very low mortality at 72 hours post-implantation (PI). In contrast, TLR2^−/−^ mice were dose-dependently susceptible to *S. aureus*, with 10^6^ cfu causing approximately 65% mortality by 72 hours PI. Somewhat unexpectedly, TLR4^−/−^ mice also showed a high mortality in response to 10^6^ cfu *S. aureus*, approaching 100% by 72 hours PI (Fig. 1a). We thus chose a dose of 10^6^ cfu *S. aureus* for all remaining experiments.

### Mitochondrial DNA

Mitochondrial DNA copy number was assessed by quantitative real-time PCR (Q-PCR) in WT and TLR2^−/−^ mice in healthy controls and at 24, 48, and 72 hours PI (Fig. 1b). The TLR2^−/−^ mice had a lower mtDNA copy number than the WT mice at all times tested, indicating either greater mitochondrial damage or a lag in mitochondrial biogenesis, or both.

### Mitochondrial Biogenesis Markers

**Figure 2:**
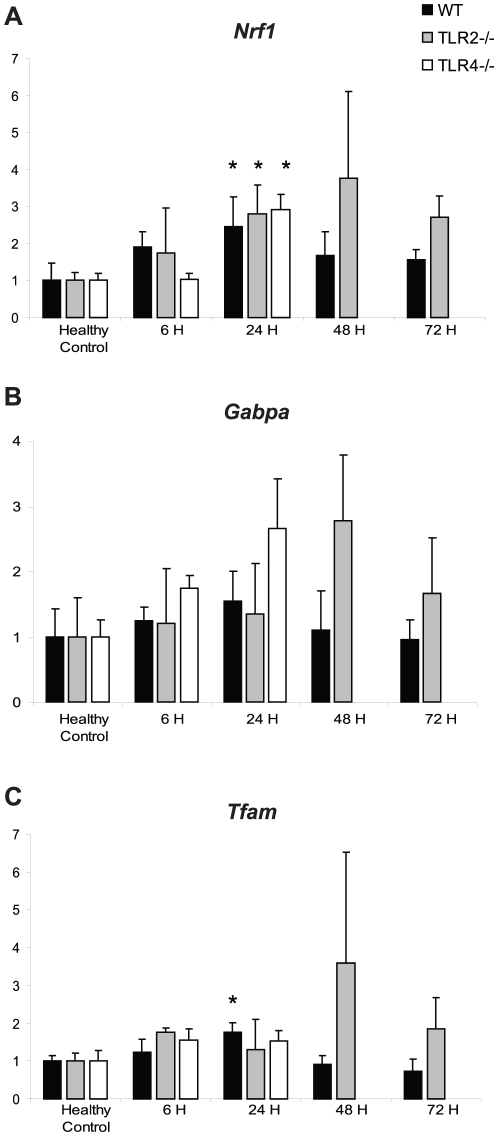
*Nrf1,Gabpa*, and *Tfam* mRNA levels in *S. aureus* sepsis. The mRNA levels of *Nrf1* (A), *Gabpa* (B), and *Tfam* (C) were measured in WT, TLR2^−/−^, and TLR4^−/−^ mice in healthy controls (HC) and at 6 h, 24 h, 48 h, and 72 h PI. n≥3 at each time point for each strain; *, p<0.05, compared to HC of the same strain.

The mRNA levels for *Nrf1*, *Gabpa*, and mitochondrial transcription factor A (*Tfam*) were measured by Q-PCR in WT, TLR2^−/−^, and TLR4^−/−^ mice (Fig. 2a, 2b, 2c). There were no between-strain statistical differences in total mRNA levels for these transcription factors. The times-to-peak, however, were different: WT mice achieved peak induction of all three transcription factors by 24 hours PI, whereas the TLR2^−/−^ mice peaked by 48 hours PI. Notably, the time course for activation for each of the three transcription factors (*Nrf1,Gabpa*, and *Tfam*) was similar, suggesting the possibility of mutual transcriptional control during acute inflammation.

**Figure 3:**
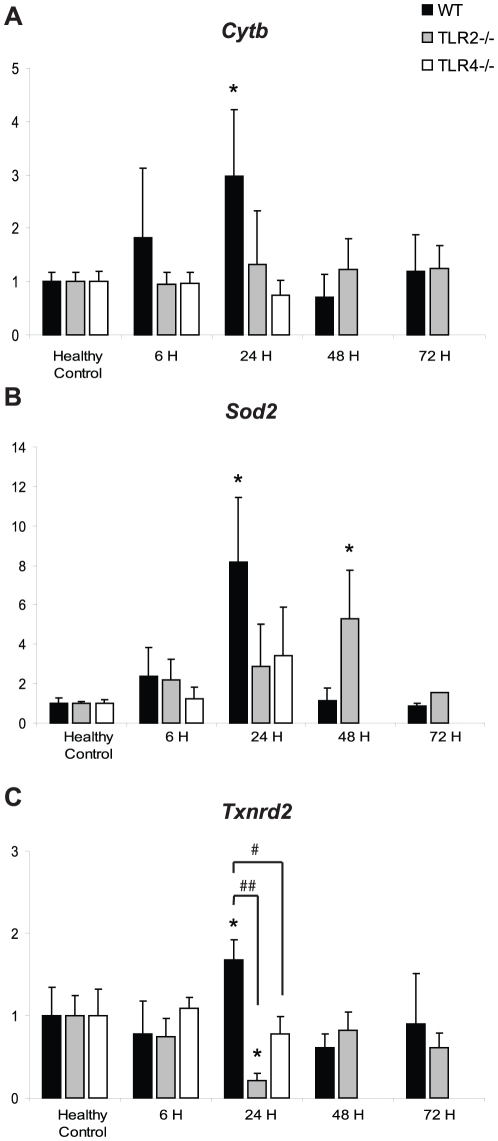
*Cytb*, *Sod2*, and *Txnrd2* mRNA levels in *S. aureus* sepsis. The mRNA levels of *Cytb* (A), *Sod2* (B), and *Txnrd*2 (C) were measured in WT, TLR2^−/−^, and TLR4^−/−^ mice in healthy controls (HC) and at 6 h, 24 h, 48 h, and 72 h PI. n≥3 at each time point for each strain; *, p<0.05, compared to HC of the same strain; #, p<0.01, ##, p<0.001 compared to 6 h time point of the indicated other strain (see further descriptive statistics in Results).

The mRNA levels of cytochrome *b* (*Cytb*), superoxide dismutase-2 (*Sod2*), and thioredoxin reductase-2 (*Txnrd2*) were measured as indices of damage and recovery of mitochondrial respiratory and antioxidant capacity, respectively (Fig. 3a, 3b, 3c). The induction pattern of each of these genes was similar in each different genetic strain. *Cytb*, *Sod2*, and *Txnrd2* were all maximally induced at 24 hours in WT mice, but the levels of *Txnrd2* were significantly lower at 24 hours in both TLR2^−/−^ and TLR4^−/−^ mice (*Txnrd2*: WT vs. TLR2^−/−^, p<0.001; WT v. TLR4^−/−^, p<0.01). *Cytb* and *Sod2* showed trends towards a decrease in the TLR2^−/−^ and TLR4^−/−^ mice compared to the WT mice (*Cytb*: WT vs. TLR2^−/−^, p = 0.1; WT v. TLR4^−/−^, p = 0.05; *Sod2*: WT vs. TLR2^−/−^, p = 0.08; WT v. TLR4^−/−^, p = 0.1). Again, this group of genes may be under mutual transcriptional control during acute inflammation, since innate immune system dysregulation impairs their transcription. Since *Cytb* is a downstream target of Tfam [25], and *Sod2* and *Txnrd2* are up-regulated in mitochondrial biogenesis [26], [27], impaired induction of the two NRF transcription factors could inhibit up-regulation of the genes for mitochondrial proteins.

### PGC family members

**Figure 4:**
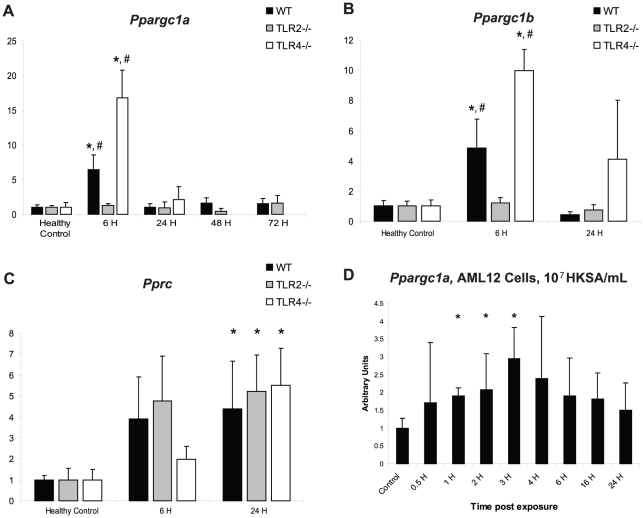
*Ppargc1a*, *Ppargc1b*, and *Pprc* mRNA levels in *S. aureus* sepsis and in cells exposed to HKSA. The mRNA levels of *Ppargc1a* (A), *Ppargc1b* (B), and *Pprc* (C) were measured in WT, TLR2^−/−^, and TLR4^−/−^ mice in healthy controls (HC) and at 6 h, 24h , 48 h, and 72 h PI. n≥3 at each time point for each strain; *, p<0.05, compared to HC of the same strain; #, p<0.05, compared to 6 h time point of the other two strains (see further descriptive statistics in Results). (D) *Ppargc1a* mRNA levels were measured in AML12 cells exposed to 10^7^ HKSA at several timepoints after exposure. *, p<0.05, compared to control cells. n = 3 independent time-course experiments.

*Ppargc1a*, *Ppargc1b*, and *Pprc* showed differential regulation in the WT, TLR2^−/−^, and TLR4^−/−^ mice (Fig 4a, 4b, 4c). *Pprc* was similarly up-regulated in all three mouse strains, with an approximate 5-fold induction at 24 hours PI (WT: 4.4-fold vs. HC, p<0.05; TLR2^−/−^: 5.2-fold vs. HC, p<0.05; TLR4^−/−^: 5.4-fold vs. HC, p<0.05). *Ppargc1a* and *Ppargc1b*, however, were both maximally induced in WT mice at 6 hours PI (*Ppargc1a*: 6.4-fold vs. HC, p<0.01; *Ppargc1b*: 4.8-fold vs. HC, p<0.05), but failed to be induced in TLR2^−/−^ mice at 6 hours PI (*Ppargc1a*: 1.3-fold vs. HC, p>0.05; *Ppargc1b*: 1.2-fold vs. HC, p>0.05), and were over-expressed in TLR4^−/−^ mice at 6 hours PI (*Ppargc1a*: 16.7-fold vs. HC, p = 0.001; *Ppargc1b*: 10.0-fold vs. HC, p<0.001). Comparisons of *Ppargc1a* and *Ppargc1b* mRNA between the three strains at 6 h show that each strain behaved significantly differently from the others (*Ppargc1a*: WT vs. TLR2^−/−^, p = 0.01; WT v. TLR4^−/−^, p = 0.01; *Ppargc1b*: WT vs. TLR2^−/−^, p<0.05; WT vs. TLR4^−/−^, p<0.01). This unexpected finding demonstrates the differential regulation of the PGC family members, with *Ppargc1a* and *Ppargc1b* showing a TLR-dependent response to *S. aureus*-induced inflammation. *Pprc* mRNA levels were not affected by deficiencies of TLR2 or TLR4, and were up-regulated at 24 h PI in all three strains. To test whether *S. aureus* directly activates *Ppargc1a* in hepatocytes, studies were conducted in AML12 cells, which were shown to express TLR2 by endpoint RT-PCR (data not shown). AML12 cells were exposed to 10^7^ heat-killed *S. aureus* (HKSA) per mL, and *Ppargc1a* mRNA levels were measured after different periods of exposure. The means of triplicate time-course experiments showed significant increases in *Ppargc1a* mRNA levels at 1, 2, and 3 hours post-exposure to HKSA (Figure 4d).

### *In silico* promoter analyses

*In silico* analyses of the proximal promoters (500 bp upstream of the transcription start site) of the three PGC family members were performed to uncover any obvious transcription factor binding site similarities between *Ppargc1a* and *Ppargc1b* that were not also present in the promoter of *Pprc* that might explain their co-regulation. Using the web-based programs zPicture and rVista, we were able to demonstrate good conservation of the *Ppargc1a* and *Ppargc1b* promoters in the mouse and the human, whereas the proximal *Pprc* promoter region is poorly conserved between mouse and human. There is modest overlap in the predicted transcription factors for the three genes, but no binding sequences that are conserved between *Ppargc1a* and *Ppargc1b* but not *Pprc* (Figure S1a). Interestingly, *Pprc* shows a high level of conservation between mouse and human in the first 1000 bp in intron 1, where there are 5 conserved binding sites for c-myc, USF, and Max. However, *Ppargc1a* has no such sites, and *Ppargc1b* shows only two (Figure S1b).

### Analysis of the 3′UTR of the PGC family members

**Figure 5:**
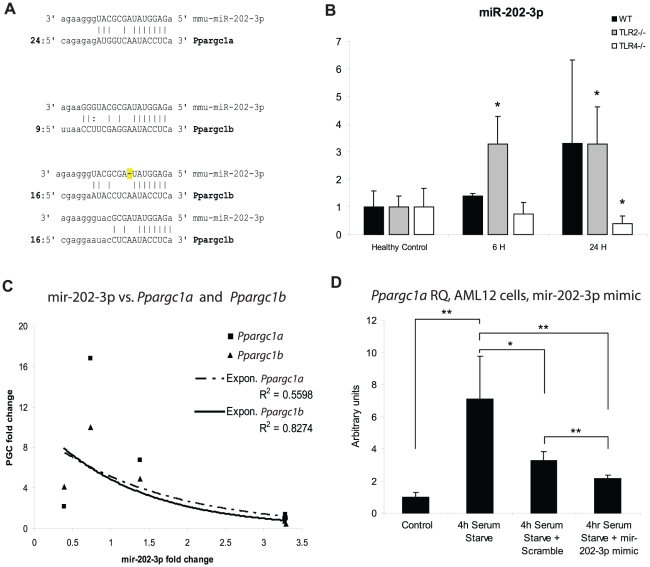
mir-202-3p is associated with *Ppargc1a* and *Ppargc1b* degradation. (A) Predicted binding of mir-202-3p to the *Ppargc1a* 3′UTR at 24 bp and to the *Ppargc1b* UTR at 9 bp and at 16 bp. (B) mir-202-3p levels were measured by Q-PCR in WT, TLR2^−/−^, and TLR4^−/−^ mice in healthy controls (HC) and at 6 h and 24 h PI. n = 3 at each time point for each strain; *, p<0.05, compared to HC of the same strain. (C) mir-202-3p expression is negatively correlated with the mRNA levels of *Ppargc1a* (R^2^ = 0.56) and *Ppargc1b* (R^2^ = 0.83). (D) AML12 cells were transfected for 24 h with either mir-202-3p mimic or scramble siRNA, and then serum-starved to induce *Ppargc1a*. mir-202-3p causes a significant decrease in *Ppargc1a* mRNA. *, p<0.05, **, p<0.01. Transfections and starvations were performed in triplicate.

Since the proximal promoters of *Ppargc1a* and *Ppargc1b* did not share any transcription factor binding sites not also shared by *Pprc*, we next examined the 3′UTRs of each gene to determine if they share binding sites for miRNAs. We found that *Ppargc1a* and *Ppargc1b* share multiple miRNA binding sites, but have none in common with *Pprc* (Figure S2). The miRNA binding sites were analyzed, and the miRNAs mmu-let-7a and mmu-mir-202-3p were predicted to have both good seed-region binding and good downstream binding to their targets in *Ppargc1a* and *Ppargc1b* (Fig. 5a). In addition, analyses of the 3′UTR seed regions performed using the online software mfold [23], [24] showed that the two genes will be in a single-strand formation in roughly one-half the predicted folding patterns, implying an availability to bind the miRNA (Figure S3).

### Mmu-mir-202-3p and PGC family genes

Specific miRNA levels were measured by Q-PCR in WT, TLR2^−/−^, and TLR4^−/−^ mice. Let-7a was tested first and showed no differential regulation among the three genetic strains (data not shown). We then tested mir-202-3p and found that it was significantly increased at 6 h and 24 h in TLR2^−/−^ mice (6 h: 3.7-fold vs. HC, p<0.001; 24 h: 3.8-fold vs. HC, p<0.01), was unchanged at 6 h, but was increased at 24 h PI with borderline significance in WT mice (6 h: 1.4-fold vs. HC, p = 0.16; 24 h: 3.3-fold vs. HC, p = 0.10), and was unchanged at 6 h but decreased at 24 h in TLR4^−/−^ mice (6 h: 1.1-fold vs. HC, p = 0.3; 24 h: 0.4-fold v. HC, p<0.05) (Fig. 5b). The miR-202-3p levels thus correlated inversely with *Ppargc1a* and *Ppargc1b* mRNA levels at 6 h and 24 h PI. To illustrate this, the average fold-inductions of *Ppargc1a* and *Ppargc1b* were plotted separately against the fold-induction of mir-202-3p at the same times in the same strains. The best-fit function showed a negative exponential relationship, with R^2^ values of 0.56 for *Ppargc1a* and 0.83 for *Ppargc1b* (Fig. 5c).

In order to confirm the association between *Ppargc1a* expression and mir202-3p under more general conditions, we obtained a microRNA mimic of mir-202-3p (a dsRNA sequence that matches the mir-202-3p sequence) for transfection into AML12 cells. AML12 cells were transfected with either mir-202-3p mimic or scrambled RNA for 24 hours, and were then serum-starved for 4 hours to induce *Ppargc1a* mRNA (See Figure 5d). In this system, *Ppargc1a* mRNA increased approximately 7-fold after starvation, but this effect was blunted by the presence of the mir-202-3p mimic (starvation only *Ppargc1a*: 7.1-fold vs. control, *P*<0.01; mir-202-3p mimic *Ppargc1a*: 2.1-fold vs. control, *P* value vs. starvation only: *P<*0.01). There was also a significant reduction from the scramble-treated cells (scramble *Ppargc1a*: 3.3-fold vs. control, *P* value vs. starvation only: *P<*0.05), however this reduction was significantly less than in the mir-202-3p treatments (scramble vs. mir-202-3p mimic *P*<0.01). The effect of scrambled RNA on the system may have been due to activation of a non-specific dsRNA response. In any event, the microRNA mir-202-3p experiment functionally confirmed a decrease *Ppargc1a* mRNA levels in cultured cells.

## Discussion

This study examined the effects of acute *S. aureus* sepsis on the expression of the PGC family and other genes that regulate mitochondrial biogenesis in WT, TLR2^−/−^, and TLR4^−/−^ mice and had two major new findings. First, there is a differential regulation of the PGC family members in *S. aureus* sepsis that occurs downstream of TLR signaling. Specifically, *Ppargc1a* and *Ppargc1b* show a peak expression at 6 h PI in WT mice (confirmed *in vitro* by exposing mouse hepatocytes (AML12 cells) to HKSA) and TLR4^−/−^ mice, whereas *Pprc* expression peaks at 24 h PI in all three strains. The fact that *Pprc* is equally expressed in all three mouse strains indicates that the gene is not being regulated by signals downstream of either TLR2 or TLR4. However, *Ppargc1a* and *Ppargc1b* gene expression are modified by changes in TLR signaling, as TLR2^−/−^ mice fail to up-regulate either gene, but TLR4^−/−^ mice show greater mRNA up-regulation than WT mice. Second, we discovered that *Ppargc1a* and *Ppargc1b* gene expression may be controlled post-translationally *in vivo*. The microRNA mir-202-3p is specific for both *Ppargc1a* and *Ppargc1b*, and its expression correlates negatively with both genes. In addition, mir-202-3p functionally decreases *Ppargc1a* mRNA levels in AML12 cells. Thus, we report that *Ppargc1a* and *Ppargc1b* are co-regulated in the acute phase of *S. aureus* sepsis by factors that are downstream of innate immune activation, and that this co-regulation is correlated with the expression of mir-202-3p.

TLR2 is activated by components of the Gram-positive cell wall, whereas TLR4 is activated in response to components of the Gram-negative cell membrane [14]. Previous studies have shown that TLR2^−/−^ mice have an increased susceptibility to *S. aureus*, and have higher bacterial loads during *S. aureus* sepsis [28]. Very few studies have examined the role of TLR4 *in vivo* in the response to live *S. aureus*. The available data show that TLR4^−/−^ mice have an impaired ability to respond to *S. aureus* [29]. Thus, our finding that TLR4^−/−^ mice respond differently than WT mice to *S. aureus* sepsis is in line with the literature. In this study, the increased mortality of TLR4^−/−^ mice (as compared to WT) in the setting of increased *Ppargc1a* and *Ppargc1b* gene expression does not rule out the possibility that overexpression of these genes could, in fact, increase survival. The loss of TLR4 has major repercussions for host defense, including impaired resolution of infection. TLR4^−/−^ mice have dysregulated immune systems such that multiple factors unrelated to mitochondrial biogenesis probably contribute to the increased mortality rate. In general, TLR-deficient mice tend to fail to upregulate inflammation early in response to their TLR-specific pathogens [30]. However, bacterial infections can activate multiple accessory pathways; for example, the receptors NOD1/2, TLR9, and DAI can all be activated in response to bacterial challenge [31], [32]. Alternative innate inflammation pathways can thus still be up-regulated in TLR-deficient mice. Overall, one limitation of this study is that is does not establish a direct link between the TLRs and *Ppargc1a* and *Ppargc1b* gene expression; instead, we showed a link between their deficiency in an otherwise intact physiologic system and differential regulation of *Ppargc1a* and *Ppargc1b*. Further studies such as *in vitro* work on TLR-deficient cells or a in a system of single TLR expression (such as TLR-expressing HEK293 cells) will be important in confirming the roles of TLRs in the regulation of *Ppargc1a* and *Ppargc1b*.

The mitochondrial biogenesis transcription factors *Nrf1,Gabpa*, and *Tfam* were all up-regulated in response to *S. aureus* sepsis in all three mouse genotypes, but there was a lag in time-to-peak in TLR2^−/−^ mice compared with WT mice (48 h vs. 24 h). In addition, TLR2^−/−^ peak transcript levels trended towards being greater than WT peak transcript levels, implying that the system attempts to rescue the TLR2^−/−^ phenotype. Furthermore, the genes of the downstream mitochondrial proteins showed less activation (*Txnrd2*) or a trend towards less activation (*Cytb*, *Sod2*) at 24 h PI in TLR2^−/−^ and TLR4^−/−^ mice compared with WT mice. Taken together, the delayed or dampened activation of the assayed genes in TLR2^−/−^ and TLR4^−/−^ mice implies that appropriate activation of mitochondrial biogenesis requires TLR signaling.

*Ppargc1a* and *Ppargc1b* were co-regulated at 6 h PI in the mouse model, so analyses of both the proximal promoter regions and the 3′UTRs of all three PGC family members were performed *in silico* in order to identify similarities that could account for the observed co-regulation. Several transcription factors are known to regulate either of the genes alone, and several microRNAs have been shown to be involved in the stability of *Ppargc1a* and other mitochondrial genes in response to exercise [16], [17], [33]. In the published literature, however, no factors have yet been reported that coordinately affect the expression of both genes. The proximal promoters of *Ppargc1a* and *Ppargc1b* showed no similarities not also shared by that of *Pprc*, but the 3′UTRs of the two genes showed binding sites for at least three of the same miRNAs, none of which were found in the 3′UTR of *Pprc*. This narrow subset of miRNAs was further restricted to those that showed good binding characteristics and whose targets in the two mRNAs were predicted to be in single-strand formation.

The microRNA mmu-mir-202 was previously shown to be one of the small subset of microRNAs that are found in the mitochondria, indicating that it may have associations with mitochondrial function [34]. Mir-202-3p was predicted to have good binding to the *Ppargc1a* and *Ppargc1b* mRNAs at sites likely to be in a single-strand formation. Measurements of mature mir-202-3p by Q-PCR revealed that mir-202-3p is increased at 6 h and 24 h in TLR2^−/−^ mice and also at 24 h in WT mice, but is decreased at 24 h in TLR4^−/−^ mice. This fits with both the lack of up-regulation of *Ppargc1a* and *Ppargc1b* mRNA in TLR2^−/−^ mice at these time points, and their return to baseline levels at 24 h in WT mice. Thus mmu-mir-202-3p expression correlates with decreased *Ppargc1a* and *Ppargc1b* in this *in vivo* model of sepsis. We suspect that this degradation is caused by binding of mir-202-3p to the identified sites in the target mRNAs, leading to their degradation by the RISC. This *in vivo* finding was confirmed by *in vitro* reduction of *Ppargc1a* in response to mir-202-3p, but the differing magnitude of the changes in *Ppargc1a* may indicate that secondary effects are also at work. MicroRNAs can individually regulate large networks of genes, so the finding that mir-203-3p correlates with the down-regulation of *Ppargc1a* and *Ppargc1b* could in theory be due to a secondary effect of mir-202-3p on a different gene. Further *in vivo* studies of the microRNA may yield additional information on the complete range of functions of mir-202-3p.

It is well-recognized that dysregulation of oxygen metabolism leads to cellular dysfunction and that mitochondrial damage correlates with mortality in sepsis. Moreover, since mitochondrial biogenesis is activated by cell survival pathways, it is a pro-survival event in sepsis. The PGC family members are known co-activators of the mitochondrial biogenesis transcription factors *Nrf1* and *Gabpa* [1], [2], [12]. Mice with embryonic deficiencies in either *Ppargc1a* or *Ppargc1b* develop minor metabolic abnormalities, but mice deficient in both factors have a severely disrupted perinatal cardiac phenotype [35]. In addition, both factors are independently capable of promoting mitochondrial biogenesis if they are over-expressed [36]. Thus, our findings that *Ppargc1a* and *Ppargc1b* are co-regulated in response to sepsis, and that mRNA stability for each correlates inversely with the expression mir-202-3p, indicate that both may serve pro-survival functions in sepsis. This also identifies mir-202-3p as a possible target for therapy, although currently there are no interventions that can target the blockade of specific miRNA *in vivo*. Future studies should identify the key transcriptional control mechanisms for mir-202-3p. This could open potential new avenues of mitochondrial therapeutics, as a decrease in mir-202-3p levels is associated with increases in *Ppargc1a* and *Ppargc1b* gene expression. This would translate to therapeutic potential if *Ppargc1a* or *Ppargc1b* gene activation could be shown to improve recovery or lessen mortality in clinical sepsis.

## Supporting Information

Figure S1 *In silico* promoter and intronal analyses of *Ppargc1a*, *Ppargc1b*, and *Pprc*. (A) The proximal 500 bp from the TSS in *Ppargc1a*, *Ppargc1b*, and *Pprc* from both mouse and human were aligned with zPicture and then submitted to rVista, with all transcription factors searched under an optimized matrix. (B) The proximal 1000 bp in intron 1 in *Ppargc1a*, *Ppargc1b*, and *Pprc* from both mouse and human were aligned with zPicture and then submitted to rVista, with all transcription factors searched under an optimized matrix.
(6.63 MB EPS)

Figure S2 *In silico* 3'UTR miRNA binding analyses of *Ppargc1a*, *Ppargc1b*, and *Pprc*. The 3'UTRs of *Ppargc1a*, *Ppargc1b*, and *Pprc* were analyzed in the mouse using TargetScan Mouse release 5.1. Shown are miRNAs with good seed-binding characteristics. The let-7 family and mir-202-3p were conserved between *Ppargc1a* and *Ppargc1b*.
(3.31 MB EPS)

Figure S3 *In silico* 3'UTR mRNA folding analyses of *Ppargc1a* and *Ppargc1b*. The entire mRNA sequences of *Ppargc1a* and *Ppargc1b* were separately fed into the program mfold, and predictions were made on average single-strand characteristics of the mRNA. Shown are the regions surrounding the predicted mir-202-3p binding sites. The green bars show the seed-binding regions, while the red bars show the length of predicted mir-202-3p binding.
(1.09 MB EPS)

Table S1 Real-time PCR primer sequences and gene accession numbers.
(0.02 MB XLS)
